# Phlegmonous colitis: another source of sepsis in cirrhotic patients?

**DOI:** 10.1186/1471-230X-9-94

**Published:** 2009-12-15

**Authors:** Thomas Holzer, Pascal Gervaz, Laurent Spahr, Thomas McKee, Pascal Bucher, Philippe Morel

**Affiliations:** 1 Department of Surgery, Geneva University Hospital and Medical School, 4 rue Gabrielle-Perret-Gentil, 1211 Geneva, Switzerland; 2 Department of Gastroenterology, Geneva University Hospital and Medical School, 4 rue Gabrielle-Perret-Gentil, 1211 Geneva, Switzerland; 3 Department of Pathology, Geneva University Hospital and Medical School, 1 rue Michel-Servet, 1211 Geneva, Switzerland

## Abstract

**Background:**

The clinical relevance of phlegmonous colitis (PC), a rare autopsy finding in cirrhotic patients, is poorly documented. We postulated that PC might be a source of sepsis in patients with portal hypertensive colopathy (PHC).

**Case presentation:**

We report three cirrhotic patients who were admitted with abdominal sepsis and who illustrate, to various degrees, the clinico-pathological sequence of colonic alterations associated with portal hypertension. Two cirrhotic patients with PHC developed gram-negative bacteraemia and quickly responded to intravenous antibiotics. Another cirrhotic patient underwent emergency colectomy for PC, and subsequently died from multiple organ failure. Histological alterations in the operative specimen included: a) mucosal ulcerations; b) disseminated micro-abscesses in the submucosa; and c) a severe vasculopathy leading to complete obliteration of submucosal blood vessels.

**Conclusions:**

These data suggest that cirrhotic patients with PHC may progress towards PC, which, in turn, may be the cause for life-threatening sepsis.

## Background

Portal hypertension diffusely affects the gastrointestinal tract, and the colon is no exception; however, portal hypertensive colopathy (PHC), which was initially described in the 1990s, is a poorly defined entity [1]. Colonic alterations associated with liver cirrhosis include a combination of vascular ectasias, mucosal oedema, rectal varices, and hyperemia [2,3]. It is estimated that vascular ectasias and rectal varices are present in up to 50% of cirrhotic patients; yet, the risk of clinically significant colonic bleeding in these patients was inferior to 6% in a prospective study with a 2-year follow-up period [4]. Thus, despite the fact that colonic lesions are frequent in cirrhotic patients, and that the fundamental pathologic change is a vasculopathy, these hemodynamic alterations are probably not associated with significant risk of lower gastro intestinal (GI) bleeding.

Phlegmonous colitis (PC) was first recognized in 1975 as a source of bacteraemia in patients with end-stage liver disease [5]. In 2009, PC remains almost exclusively an autopsy finding, and was actually detected *post-mortem *in 2.5% of patients with cirrhosis: thirteen autopsy cases showed some or all of the following clinico-pathologic characteristics: (1) preferential involvement of the cecum; (2) phlegmonous changes in the submucosa; and (3) bacterial infection [6,7]. These results suggest that PC is a severe and specific complication of portal hypertension. We postulated that, in parallel with aggravating portal hypertension, PHC could progress towards PC and sepsis. We report herein three patients, who illustrate this sequence of events.

## Case Presentation

All three patients were admitted in our institution for abdominal pain and underwent a CT-scan evaluation, which demonstrated a colonic inflammation. We report herein the three patients in increasing order of septic complications. Additional file 1: Table S1 summarizes the results of various microbiologic analyses performed in these three patients.

### CASE 1

**Figure 1:**
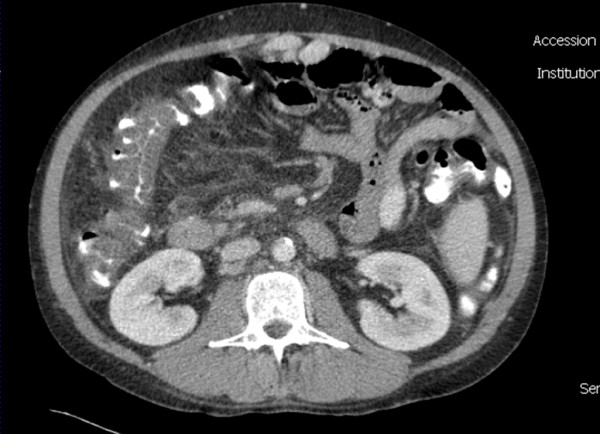
**CT scan of case 1 showing extensive oedema of the right a transverse colon, as well as cirrhotic alterations of the liver**.

A 43 year-old alcoholic male presented at the Emergency Department with diffuse abdominal pain. The patient had been previously diagnosed with Child C cirrhosis, and suffered one prior episode of upper GI bleeding. On admission, the patient was hemodynamically stable, with a distended and diffusely painful abdomen, but no signs of peritonitis. Blood tests showed a mild inflammatory syndrome. CT scan revealed a liver with cirrhotic aspect, as well as all classical signs of portal hypertension, including ascites, splenomegaly and repermeabilization of the umbilical vein. There was a marked thickening of the large bowel, predominantly on the ascending and transverse colon (Figure 1). Hemocultures were positive for *Enterococcus faecalis*, and the initial antibiotic regimen, consisting of ceftriaxone and metronidazole IV, was replaced by ertapenem 1 × 1 G/day IV. The patient did well and was discharged home ten days after his admission.

### CASE 2

**Figure 2:**
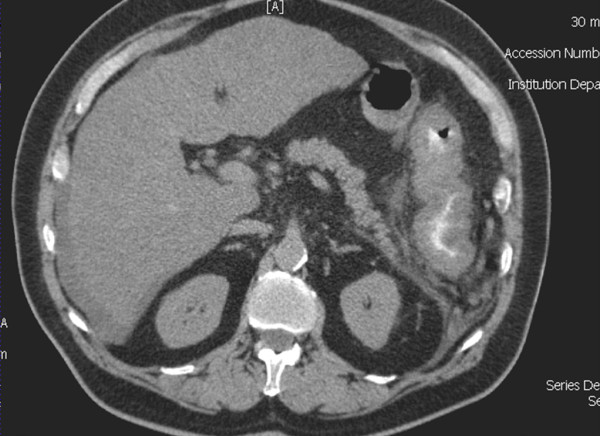
**CT scan of case 2 showing again extensive inflammation of the entire colon (pancolitis), with oedema and thickening of the large intestine wall**.

A 70 year-old male presented at the Emergency Department with acute abdominal pain and hypotension. Medical history was positive for Child B cirrhosis, hepatitis C, oesophageal varices, and diabetes; he previously suffered one episode of right colitis of unknown origin that responded well to a treatment of ceftriaxone and metronidazole. On admission, the patient was acutely ill, with a severe inflammatory syndrome, and was transferred to the Intensive Care Unit for resuscitation. CT scan showed a cirrhotic liver, ascites in low quantity, and an aspect of pancolitis with a thickened and oedematous wall (Figure 2). Hemocultures were positive for *Enterococcus *and *Streptococcus bovis*. The patient was treated with a combination of amoxicilline-clavulanic acid and gentamicine, with rapid improvement in both clinical and biological signs of inflammation. The patient was discharged on day 15 after his admission.

### CASE 3

**Figure 3:**
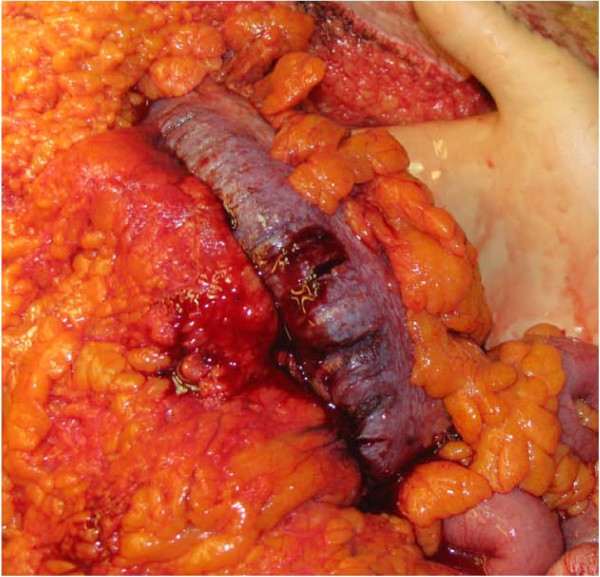
**Intra-operative aspect of the ascending colon of case 3**. The colon was indurated, congestive, thickened, the general appearance being somewhat similar to acute mesenteric vein thrombosis.

**Figure 4:**
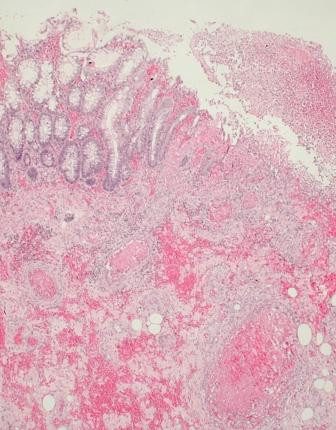
**Histological features of the operative specimen, showing numerous micro-abscesses in the submucosa (asterisk) as well as deep mucosal ulceration (arrow)**.

**Figure 5:**
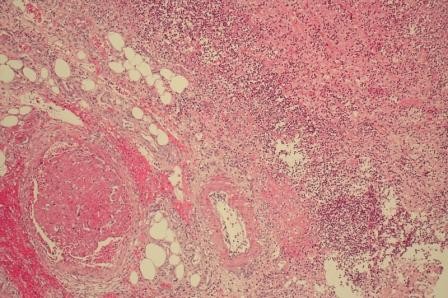
**Histological features of the operative specimen, showing in addition to confluent micro-abscesses in the submucosa, a severe vasculopathy with disseminated venous thrombosis**.

A 69-year-old alcoholic male was admitted for abdominal pain, associated with diarrhoea and hematochezia. The patient was also known for cirrhosis and type II diabetes. On admission, the patient was in poor condition and presented with peritonitis associated with hypotension, acute renal failure and sepsis. The patient was transferred to the intensive care unit for resuscitation and IV antibiotics (ceftriaxone and metronidazole) were administered. CT scan of the abdomen showed, in addition to cirrhotic liver, a marked thickening of the whole colon with infiltration of the peri-colic fat, and ascites in low quantity. 24 hours after admission, the patient developed increased abdominal pain associated with a septic shock and was taken to the operating room. At surgery, the colon was thick, indurated and congestive; lesions predominated within the ascending colon (Figure 3). A total colectomy with terminal ileostomy and a hepatic biopsy were performed. Histopathology examination of the surgical specimen revealed: 1) disseminated erosions/ulcerations of the colonic mucosa (Figure 4); 2) multiple micro-abscesses located in the sub-mucosa in association with polynuclear infiltration, and 3) a vasculopathy characterized by venous ectasias and numerous venous thrombi (Figure 5). The hepatic biopsy showed an important cirrhotic alteration of the parenchyma with fresh thrombosis of the branches of the portal vein. One week after surgery, the patient developed multiple organ failure and died. Preoperative culture of ascites came back positive for *Pepto-streptococcus*.

## Conclusion

Cirrhotic patients are particularly susceptible to bacterial infections and the origin of these microorganisms (most commonly *Escherichia Coli, Enterococcus faecalis, Proteus mirabilis *and *Pseudomonas aeruginosa*) is often the lower digestive tract. Most clinicians would concur that: 1) the colon is the source of bacteraemia in cases 1 and 2; and 2) bacterial translocation due to increased intestinal permeability is the most likely physiopathology mechanism involved. However, bacterial translocation has been mostly documented in small animals [8], and is actually rare in Child A (3%) and B (8%) patients. In summary, both patients developed abdominal pain and bacteraemia; blood cultures were positive for enteric microorganisms, the colon (predominantly on the right side) was abnormal with radiologic evidence of severe oedema or inflammation of the colon and no other possible source of sepsis was found (Additional file 1).

We hypothesized that PHC may progress towards PC. The latter condition, which requires a biopsy or a surgical specimen is rarely (if ever) documented *ante mortem*. Another fatal case of phlegmonous colitis has been recently reported in a cirrhotic patient receiving a combination of interferon and ribavirin for hepatitis C [9]. We suspect that this entity goes often unrecognized, because the patients' poor condition precludes any surgical intervention. Interestingly, in case 3, the only positive culture was the presence of bacteria in ascites, and it is likely that, in the absence of an operative specimen, the final diagnosis would have been spontaneous bacterial peritonitis (SBP) due to intestinal translocation. We consider that this patient had infected ascites indeed, but bacterial peritonitis in that case was secondary to phlegmonous colitis.

The data presented here suggest that in some cirrhotic patients with portal hypertensive colopathy, phlegmonous colitis could be the origin of sepsis. The underlying mechanism is a worsening of vasculopathy leading to complete obliteration of submucosal blood vessels. Mucosal erosions secondarily appear and are responsible for disseminated micro-abscesses formation in the submucosa, which constitutes the probable source of gram-negative sepsis. PC and PHC are closely related as suggested by the fact that both conditions share similar histological features: specifically, vascular alterations described in PHC (dilated venules and vascular ectasias in the submucosa) are present in PC. By contrast, mucosal ulcerations and the presence of microabscesses are dominant features of PC, which are absent in PHC. In conclusion, the three cases presented herein suggest that portal hypertensive colopathy could sometimes progress towards phlegmonous colitis. Whilst portal hypertension-induced vascular changes are initially similar throughout the GI tract, the clinical manifestations are eventually dominated by bleeding for the upper GI, and sepsis for the colon.

## Abreviations

PHC: portal hypertensive colopathy; GI: gastro intestinal; PC: phlegmonous colitis.

## Competing interests

The authors declare that they have no competing interests.

## Consent

Consent forms signed by the patients' relatives are submitted separately

## Authors' contributions

**TH: **protocol design, editing manuscript. **PG: **protocol design, editing manuscript. **LS: **protocol design, editing manuscript. **TMK: **tissues processing, histological analysis of surgical specimen. **PB: **surgical operation, editing manuscript. **PM: **final review, supervision of scientific content of manuscript. All authors have read and approved the final version of manuscript.

## Pre-publication history

The pre-publication history for this paper can be accessed here:

http://www.biomedcentral.com/1471-230X/9/94/prepub

## Supplementary Material

Additional file 1 **Summary of Microbiology Analyses**. Results of blood and ascites analyses performed in the three patients. Click here for file
